# Real‐Time Cellular Cytochrome C Monitoring through an Optical Microfiber: Enabled by a Silver‐Decorated Graphene Nanointerface

**DOI:** 10.1002/advs.201701074

**Published:** 2018-06-07

**Authors:** Hongtao Li, Yunyun Huang, Chaoyan Chen, Aoxiang Xiao, Guanhua Hou, Yugang Huang, Xinhuan Feng, Bai‐Ou Guan

**Affiliations:** ^1^ Guangdong Provincial Key Laboratory of Optical Fiber Sensing and Communications Institute of Photonics Technology Jinan University Guangzhou 510632 China; ^2^ School of Pharmaceutical Sciences Guangzhou Medical University Guangzhou 511436 China

**Keywords:** Ag@RGO interfacial sensitization, apoptosis monitoring, cytochrome c, optical microfiber, real time detection

## Abstract

The translocation of cytochrome c (cyt c) from mitochondria and out of cell is an important signal of cell apoptosis. Monitoring this process extracellularly without invasion and cytotoxicity to cells is of great importance to understand certain diseases at the cellular level; however, it requires sensors with ultrahigh sensitivity and miniature size. This study reports an optical microfiber aptasensor with a silver‐decorated graphene (Ag@RGO) nanointerface for real‐time cellular cyt c monitoring. Owing to an interfacial sensitization effect coupled with the plasmonic electromagnetic enhancement of silver nanoparticles and chemical enhancement of graphene platforms, which enhances the energy density on microfiber surface obviously, the lowest limit of detection achieved is 6.82 × 10^−17^ m, which is approximately five orders of magnitude lower than those of existing methods. This microfiber successfully detects the ultralow concentrations of cyt c present during the initial stage of apoptosis in situ. As the microfiber functionalized by Ag@RGO nanointerface can be varied to meet any specific detection objective, this work opens up new opportunities to quantitatively monitor biological functions occurring at the cellular level.

## 1 Introduction

Cytochrome c (cyt c), a multi‐functional enzyme, is involved in both life and death decisions of cells.^1^ In the apoptosis, cyt c releases from mitochondria, and then releases from cells as the cells break down. Its release is an essential signal of the initial progression of programmed cell death—apoptosis.^2^ In clinical disease diagnosis, once pathological conditions are triggered by cardiac failure, DNA damage, cytoskeleton disruption, or invasion from outside cells, the cyt c in mitochondria can be released into the bloodstream via permeabilization of injured mitochondria.^2^, ^3^, ^4^ Thus, cyt c monitoring not only serves as an apoptosis biomarker, providing valuable information about the nature and extent of this process, but also is of great importance to understand certain diseases at the cellular level.^5^, ^6^, ^7^ The techniques available currently in laboratories to measure cyt c release include flow cytometry,^2^ Western blot,^2^ enzyme‐linked immunosorbent assay,^4^ high‐performance liquid chromatography,^2^ and spectrophotometry.^6^ Despite their promise, these techniques might suffer from the expensive instruments, time‐consuming sample preparation protocols, and expertise needed for operation.^2^ The lowest limit of detection (LOD) among them is only 10^−12^ m,^2^ which seems insufficient to trace the initiation of apoptosis in situ. Transition of clinical laboratory cyt c assays to point‐of‐care settings, which require assays with high sensitivity, low LOD, miniature size, and cost‐effectiveness, could provide therapists and biologists with timely diagnostic details at the cellular level to make appropriate decisions regarding diagnosis and treatment.^8^, ^9^ Therefore, the development of this approach is a trend in cyt c sensors and challenge for researchers.

Recently, some exciting cutting edge technologies for cyt c detection have been studied and reported.^10^, ^11^ Wang and his co‐workers^10^ demonstrated a novel upconversion@polydopamine core@shell nanoparticle based aptameric biosensor for biosensing and imaging of cyt c inside living cells. Its high cellular internalization efficiency and low cytotoxicity made it a wonderful tool for the qualitative detection of cyt c concentration changes in complex biological matrices and monitoring cyt c mediated cell apoptosis pathway, although the sensitivity of it might need to be further improved to meet the demand of detecting cyt c in the early stage of apoptosis.

**Figure 1:**
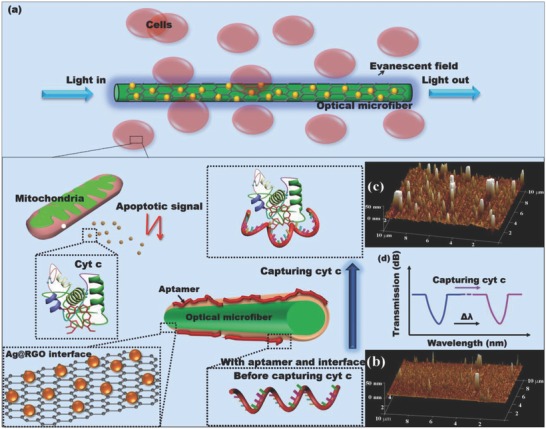
a) Schematic of the process of detecting cyt c by an optical microfiber. b) Atomic force microscopy (AFM) image of the microfiber surface before capturing cyt c (with aptamer and interface but without cyt c). c) AFM image of the microfiber surface after capturing cyt c (scale: 10 µm × 10 µm × 50 nm, the morphology effect of silica fiber base has been removed). d) Capturing cyt c induced an immense shift in the interferometric fringe in the transmission spectrum.

Herein, as an alternative approach to cellular cyt c monitoring, an optical microfiber aptasensor enhanced with a silver nanoparticle (Ag NP) functionalized graphene nanointerface has been developed. Optical microfibers are effective for real‐time quantitative analysis of the adsorption of molecules on their surface, which can modulate their surface refractive index (RI).^12^ Here, a microfiber with a diameter of 10.5 µm is placed in the clusters of cells (**Figure** **1**a). This extracellular measurement method avoids the invasion and cytotoxicity to cells. In the apoptosis, when cyt c is released from the cell, the capture of cyt c by aptamers on the optical microfiber surface (Figure 1a) causes changes in surface roughness and thickness (Figure 1b,c). Figure 1b shows the surface of microfiber with interface and aptamer before capturing the cyt c (the complete sensor without cyt c). Figure 1c shows the surface of microfiber after capturing the cyt c (the complete sensor with cyt c). It is observed that after capturing the cyt c, the surface roughness and thickness of microfiber increases obviously, and therefore varied the surface RI. The surface RI information is translated into an optical signal (Figure 1d). Owing to its advantages of miniature size (i.e., on the micrometer scale), light weight, portability, short response time, electromagnetic immunity, and mechanical flexibility, optical microfibers are suitable for integration with current analytical tools for sensing in inaccessible locations. They provide great potential for in situ monitoring and analysis in a real matrix.^13^ For the reason that the cyt c first releases out of mitochondria into the narrow space inside the cell, and then into the extracellular environment, the sensor detecting cyt c extracellularly in the early stage of apoptosis requires a lower LOD than that of the intracellular sensor. To improve the sensitivity and lower the LOD to meet the requirement of in situ cell monitoring, we have constructed a nanointerface with Ag NP‐functionalized reduced graphene oxide (Ag@RGO) between silica microfiber and aptamers (Figure 1a). As a result of the plasmonic electromagnetic field enhancement of Ag NPs,^14^ as well as the accelerated electron transformation^15^, ^16^ and electronic characteristics^17^ of reduced graphene oxide (RGO), the LOD of the as‐prepared sensor achieved is 6.82 × 10^−17^ m, which is five orders of magnitude lower than those of existing electrochemical methods (5.0 × 10^−12^ m),^18^ six orders of magnitude than those of surface plasmon resonance arrays (5.0 × 10^−11^ m),^19^ and nine orders of magnitude than both fluorescence methods (2.0 × 10^−8^ m)^20^ and optical microfiber methods without Ag@RGO nanointerface. Such a low LOD meets the requirement of in situ monitoring of the ultralow concentrations of cyt c present outside the cells during the initial step of apoptosis. Therefore, the as‐prepared improved LOD sensor paves the way for early‐stage analysis of organ pathology at the cellular level, without invading or poisoning cells.

## 2 Results and Discussion

### 2.1 Functionalization of Ag@RGO Nanointerface

**Figure 2:**
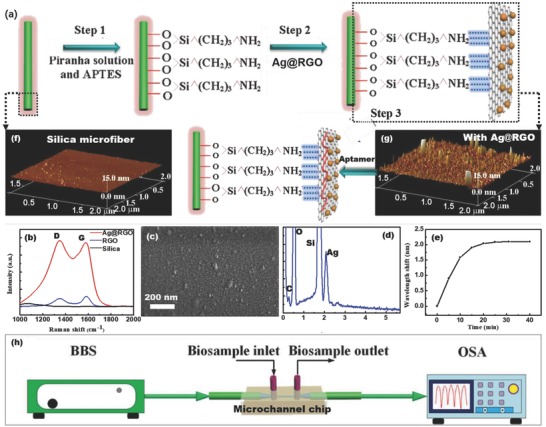
a) Schematic of the functionalization of microfiber probe. b) The Raman spectra of microfiber surfaces. c) SEM image and d) EDX of microfiber with Ag@RGO nanointerface. e) The wavelength shift of interferometer in the Ag@RGO nanointerface functionalization process. f) AFM image of the naked silica microfiber surface. g) AFM image of the microfiber surface functionalized by Ag@RGO. h) Schematic of the optical setup for monitoring cyt c.

The silica optical microfiber with a waist diameter of 10.5 µm (Figure S1a,b, Supporting Information) was fabricated by a flame‐brushing technique as shown in Figure S2 (Supporting Information).^21^ Its taper structure excites the higher‐order mode (HE_12_), which interferes with the fundamental mode (HE_11_), creating an interferometric fringe in the fiber transmission spectrum (Figure S3, Supporting Information).^22^, ^23^ As the HE_12_ mode spreads into the surface coating (Figure S4, Supporting Information), it senses the surface RI change and translates it into a wavelength shift in the interferometric fringe.^12^, ^24^ The surface of the silica microfiber was aminated by sequential modifications with piranha solution and 3‐aminopropyl‐triethoxysilane (APTES) solution (**Figure** **2**a), resulting in a surface rich in positive charge.^14^, ^25^ The Ag@RGO nanosheets (The silver nanoparticles were chemically bonded with the RGO nanosheets.^26^, ^27^, ^28^), as shown in Figure S5 and S6a in Supporting Information, were functionalized onto the silica microfiber surface by the electrostatic attraction between the remaining oxygen containing groups on RGO^29^ and the amino groups on the microfiber. (The Raman spectrum in Figure 2b, scanning electron microscopy (SEM) in Figure 2c, and energy dispersive X‐ray (EDX) in Figure 2d and Figure S7 (Supporting Information) taken on the as‐prepared microfiber surface demonstrate the successful construction of the Ag@RGO nanointerface on the silica microfiber; the Raman spectra in Figure 2b also indicate an obvious surface‐enhanced Raman scattering on RGO surface after being conjugated with Ag NPs.) In this process, a transformation from smooth (Figure 2f: the atomic force microscopy (AFM) image of silica microfiber surface without any functionalization) to rough (Figure 2g: the AFM image of microfiber surface functionalized by the Ag@RGO nanointerface) occurred on the microfiber surface, reflected by the optical spectrum as gradual redshift and leveling off as shown in Figure 2e. Subsequently, single‐stranded DNA (ssDNA) aptamers were conjugated on the Ag@RGO surface by *π–π* stacking interactions (the S element on microfiber surface in the EDX mapping shown in Figure S8 (Supporting Information) proves the successful immobilization of the aptamers.).^30^ Thus, the microfiber probe was ready for cyt c monitoring. In order to realize a single‐layer and uniform nanointerface, Ag@RGO dispersions with different concentrations have been employed in the nanointerface functionalization process. As shown in Figure S6 (Supporting Information), the Ag NPs diameter of Ag@RGO was ≈15 nm. It can be observed in Figure S9 (Supporting Information) that, when functionalized by a dispersion with low concentration (0.02 g L^−1^), there was not enough quality of nanosheets to form a single layer nanointerface (Figure S9a, Supporting Information). On the contrary, when using dispersions with higher concentrations (0.08, 0.10, and 0.12 g L^−1^), obvious aggregations appeared on the interface (Figure S9c–e, Supporting Information). When using dispersion with a concentration of 0.05 g L^−1^, a single‐layer and uniform nanointerface were observed as shown in Figure S9b (Supporting Information). To maintain stability during biotesting, a microchannel chip was employed in the experiments as shown in Figure 2h. The sensor was fixed in the microchannel (width 200 µm × height 1.5 mm) with the help of UV‐sensitive adhesive both sides over the sensing element of centimeters in length (for a total sensing volume of ≈500 µL). The testing solutions were injected into the microfluidic chip via an electronic‐controlled pump. This microchannel chip provided an airtight environment for the microfiber measurement, and eliminated the potential environmental influence (e.g., temperature fluctuations) during the biosample measurement.

### 2.2 Detection of Cyt c Solution

**Figure 3:**
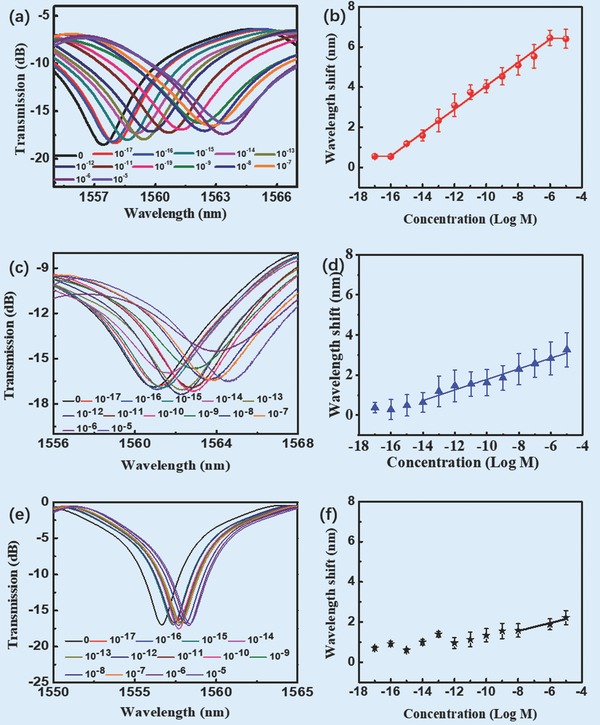
a,c,e) The measured transmission spectra of the as‐prepared sensor and control microfibers in the presence of cyt c. b,d,f) The wavelength shifts of the as‐prepared sensor and control microfibers versus the concentration of cyt c. ((a) and(b) the as‐prepared sensor; (c) and (d) the sensor functionalized with the GO nanointerface; and (e) and (f) the sensor without any interface.)

The cyt c detection ability of the as‐prepared optical microfiber aptasensor was investigated in this microchannel chip, along with control experiments. Because the silica microfiber lacks sensitivity and selectivity for cyt c (Figure S10, Supporting Information), the role of the nanointerface on silica microfiber is estimated here. When exposed to solutions of cyt c with concentrations from 0 to 10^−5^ m, the sensor showed optical spectrum modulation, as presented in **Figure** **3**a. As the cyt c concentrations increased from 10^−17^ to 10^−6^ m, an obvious redshift of transmission notch with a sensitivity of 0.583 nm per log m and a linearity of 99.7% was recorded as shown in Figure 3b. Notably the LOD of this sensor was 6.82 × 10^−17^ m, which was five orders of magnitude lower than those of existing cyt c detection methods (≈5.0 × 10^−12^ m).^18^, ^31^, ^32^, ^33^, ^34^ Such an LOD could facilitate trace analysis of cyt c. As control experiments, a microfiber aptasensor functionalized by GO nanointerface without Ag NPs (Figure 3c,d) and a microfiber aptasensor without any interface (Figure 3e,f) were employed to evaluate the effect of the Ag@RGO nanointerface on sensing. These control sensors clearly exhibited poorer sensing performances as shown in **Table** **1**. Compared to the aptasensors with only GO nanointerface and without any interface, the as‐prepared aptasensor showed a sensitivity two times higher and four times higher, respectively, and an LOD three orders of magnitude lower and nine orders of magnitude lower, respectively. Clearly, the Ag@RGO nanointerface played a great role in the sensitivity improvement.

**Table 1:** Sensing abilities of the Ag@RGO‐functionalized aptasensor and control aptasensors

Aptasensor with nanointerface	LOD [m]	Sensitivity [nm per log m]
Ag@RGO	6.82 × 10^−17^	0.583
GO	2.40 × 10^−14^	0.265
None	1.88 × 10^−8^	0.160

### 2.3 Enhancement of the Ag@RGO Nanointerface

**Figure 4:**
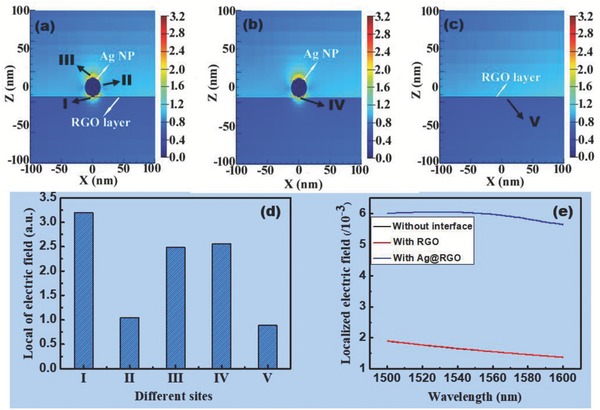
The calculated near‐field intensity mapping of the microfiber surface functionalized by a) Ag@RGO, b) Ag NPs, and c) RGO nanointerface. d) The local intensity of the surface electric field at different sites from (a)–(c). e) The localized electric field of the different nanointerfaces over an NIR wavelength range of 1500–1600 nm.

**Figure 5:**
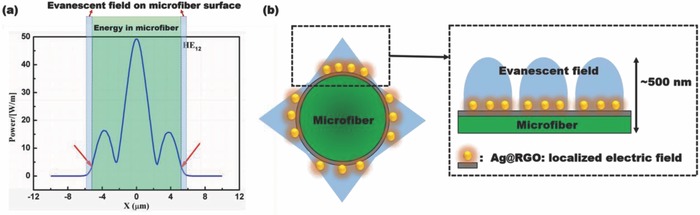
a) The transverse electric field amplitude distributions of HE_12_ mode of the silica microfiber. b) Schematic of the surface electric field enhancement mechanism by the localized electric field of Ag@RGO nanointerface.

To explore the enhancement effect of the Ag@RGO nanointerface, the near‐field intensity mapping and localized electric field over the near‐infrared (NIR) wavelength range of 1500–1600 nm of different nanointerfaces were calculated by a finite‐difference time‐domain method (**Figure** **4**). Figure 4a–c visually presents the near‐field intensity distribution on the surface of the different nanointerface‐functionalized microfibers. The intensity of the electric field between the RGO and Ag NPs on Ag@RGO nanointerface was higher than those at other Ag NP sites on this nanointerface, and higher than that of Ag NPs without RGO support, and much higher than that of the RGO surface without Ag NPs (Figure 4d). Accordingly, the power of plasmonic electromagnetic field with Ag@RGO nanointerface was much higher than those with only the RGO nanointerface and without any nanointerface. Figure 4e shows that the electric field enhancement of the Ag@RGO‐functionalized microfiber at ≈1560 nm was approximately four times higher than that of the microfiber without any interface. To explain the enhancement mechanism, a numerical mode simulation (COMSOL) software was used to analyze the transverse electric field amplitude distributions of the HE_12_ mode as shown in **Figure** **5**a. It reveals that for a microfiber with a diameter of 10.5 µm, most of the energy was concentrated within the core, and only a little of it evanesced out as the evanescent field.^35^ The evanescent field was on the microfiber surface, and could interact with the surroundings.^35^ Based on this, the surface RI change could modulate the optical spectrum. In our case, the penetration depth (the distance to which the evanescent field extends beyond the fiber surface) was ≈500 nm (Figure 5). The enhancement of this part of energy will significantly enhance the interaction between light and surroundings, and therefore greatly improves the perception ability of the optical fiber.^35^ Here, with a thickness of ≈15 nm (according to the AFM image in Figure 2g), the Ag@RGO nanointerface overlapped with the evanescent field of microfiber, causing its localized electric field to overlap with the evanescent field (Figure 5b). Therefore, by interacting with the evanescent field of the optical microfiber, the plasmonic electromagnetic field of Ag NPs on nanointerface clearly increased the energy density at the microfiber surface.^13^, ^14^, ^36^, ^37^ Because the sensitivity of optical microfiber is proportional to the surface binding‐induced change in propagation constants (Δβ) and Δβ is proportional to the evanescent field of microfiber (*E*), as expressed by Equation (1),^38^ the sensitivity is proportional to the surface energy of the microfiber^12^

(1) $$\mathrm{Sensitivity}\;=\;k_{1}•\Delta \beta \;=\;k_{2}•E\left(x,y\right)$$

where *k*_1_ and *k*_2_ are constant coefficients.

In the case of the microfiber with Ag@RGO nanointerface, the surface evanescent field was enhanced by the localized electric field of Ag NPs, which made the sensor more sensitive to the cyt c capture‐induced RI change.^39^, ^40^, ^41^

On the other hand, the RGO platform also contributed to the improvement in sensing performance. As widely employed in many electrochemical sensors,^15^, ^16^, ^17^, ^42^, ^43^, ^44^, ^45^ RGO with a partial planar structure and abundant oxygen‐containing functional groups provided a suitable platform (high surface area^43^ and more binding sites^45^) for aptamer immobilization by *π–π* interactions in this work.^30^ Simultaneously, slight changes in the charge environment of the RGO sheet arising from the binding of cyt c may result in an improved change in electronic characteristics,^46^ which sharpened the sensitivity of sensor. Moreover, in Figure 4d, the Site I of Ag@RGO (the interface between Ag NPs and RGO) was higher than the other sites, including the other sites of Ag@RGO (Sites II and III), and surface of Ag NPs without RGO support (Site IV), and also the surface of RGO without Ag NPs (Site V). It has been reported that, as an efficient acceptor of plasmonic hot electrons from Ag NPs,^47^ the RGO nanosheets enhanced the localized electric field in the nanointerface between the RGO and Ag NPs. It explains the stronger localized electric field observed on the Ag@RGO than just Ag NP particles. Therefore, the coupling of the RGO platform with Ag NPs enhanced the sensitivity of the as‐prepared sensor, enabling its detection of trace concentrations of cyt c.

### 2.4 Selectivity of Sensor

**Figure 6:**
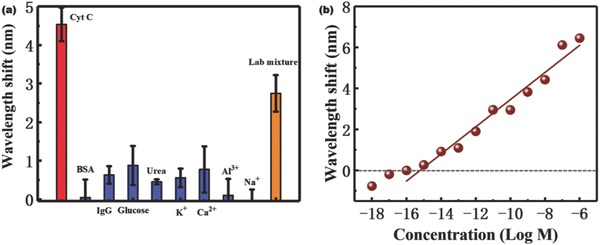
a) Comparison of optical response of sensor to 10^−9^ m pure cyt c solution, and other potential interferents with high concentration (10^−6^ m), and the lab mixture of 10^−9^ m cyt c, 10^−6^ m BSA, 10^−6^ m IgG, 10^−6^ m glucose, and 10^−6^ m Ca^2+^. b) The wavelength shifts of the as‐prepared sensor and control microfibers versus the concentration of cyt c in the presence of 10^−6^ m glucose.

Although the sensor aims at an extracellular detection, the interfering molecule fluctuations in the extracellular environment are the inevitable factor in our real detection environment, which should be considered and discussed in the detection condition. To reflect the physiological conditions in real clinical measurements, the ability of the sensor to distinguish low‐concentration of cyt c (10^−9^ m) from other high‐concentration interference components (for example, bovine serum albumin (BSA), immunoglobulin G (IgG), glucose, urea, and K^+^, Ca^2+^, Al^3+^, and Na^+^ with high concentration of 10^−6^ m) that might be present was investigated in **Figure** **6**a. This experiment showed that only cyt c induced a dramatic optical signal response (≈4.5 nm wavelength shift), whereas the other substances induced no significant optical response. Furthermore, in the mixture with low‐concentration cyt c (10^−9^ m) and high‐concentration interfering molecules (BAS, IgG, glucose, and Ca^2+^ with concentrations of 10^−6^ m), the sensor also displayed an obvious optical signal response, which could be attributed to the presence of cyt c as shown in Figure 6a (≈3 nm wavelength shift). It was slightly lower than that in the pure cyt c solution because there might be some energy loss caused by such high concentrations of interfering molecules.^48^

In order to further explore the sensor‘s potential in real‐world application, its cyt c detection ability in the presence of at least one interferent (glucose of 10^−6^ m was chosen here) was investigated (Figure 6b). As the cyt c concentrations increased from 10^−16^ to 10^−6^ m, an obvious redshift of transmission notch with a sensitivity of 0.660 nm per log m and an LOD of 4.54 × 10^−15^ m was recorded. This LOD was slightly higher than that in the pure cyt c solutions. However, it still seems low enough for in situ application. Therefore, the other ion concentration fluctuations could not generate the obvious signals or noises during our monitoring. The selectivity paves the way for in situ cell monitoring.

### 2.5 Monitoring Cyt c in Cells

**Figure 7:**
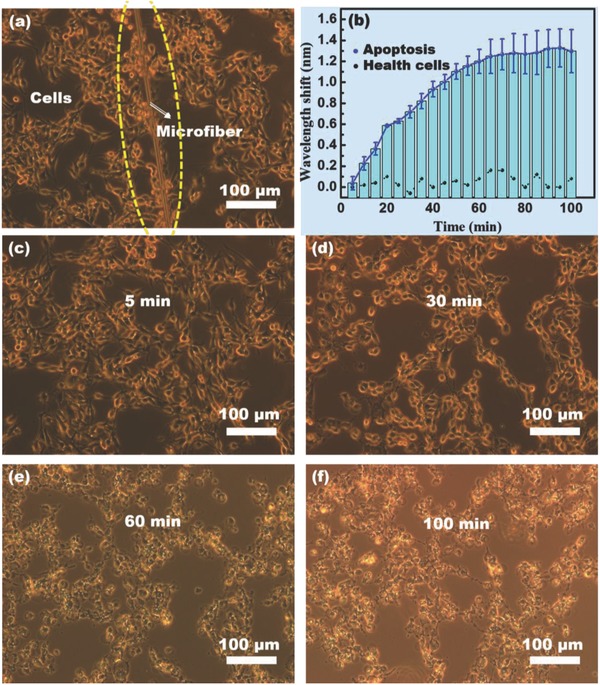
a) Optical microscopy image and b) measured wavelength shift with H_2_O_2_ (blue solid line) and without H_2_O_2_ (black dotted line) of cyt c monitoring in cells in the apoptosis tracing. c–f) Optical microscope photos of different time stages of the cells in the apoptosis process.

**Figure** **7** demonstrates the capability of the as‐prepared microfiber to monitor cyt c in cells. Mouse melanoma cells (B16‐F10) were employed as shown in Figure 7a. The micrometer‐sized footprint of microfiber enables its easy implantation in sensing in cells. When the microfiber was used for sensing in healthy cells (not adding H_2_O_2_ to accelerate apoptosis), no obvious optical signal was recorded in 100 min (Figure 7b). When 8 µL of H_2_O_2_ was added into the culture medium to accelerate apoptosis, there was still no obvious optical signal in the first 5 min, whereas the wavelength shifts increased dramatically from 5 to 60 min and then leveled off gradually after 60 min. This result indicates that the microfiber perceived the cyt c concentration increasing from 5 to 60 min and stabilizing after 60 min. The corresponding cell morphology was captured by an optical microscope, as shown in Figure 7c–f. In the first 5 min after H_2_O_2_ injection, the cell morphology was indistinguishable from that of healthy cells, indicating there was no obvious apoptosis. At 30 min, a few cells were observed to start severing attachments to other cells and the extracellular matrix, and to rounded up (Figure 6d), indicating that a few of the cells were dying.^49^, ^50^ At 60 min, a large number of cells with morphological signs of death appeared (Figure 7e). In addition, the number of dying cells at 100 min was similar to that at 60 min (Figure 7f). These results demonstrated that the cell morphology corresponded to the wavelength shift at the same time. Therefore, the as‐prepared microfiber can monitor the cyt c concentration and apoptotic process in cells in real time.

## 3 Conclusion

In conclusion, this work presented an optical microfiber aptasensor with a Ag@RGO nanointerface for real‐time cellular cyt c monitoring. Owing to an interface‐induced sensitization effect coupled with the use of silver nanoparticles and graphene platforms, the LOD achieved is 6.82 × 10^−17^ m, which is at least five orders of magnitude lower than those of existing methods. The ultralow LOD and miniature size of the sensor enabled the in situ detection of the ultralow concentrations of cyt c present during the initial stage of apoptosis. As the microfiber functionalized by the Ag@RGO nanointerface can be varied to meet any specific detection objective, this work opens up new opportunities to quantitatively monitor biological functions occurring at the cellular level.

## 4 Experimental Section

*Silica Optical Microfiber Sensor*: The silica optical microfiber structure fabrication was as previously described.^22^, ^23^ In brief, by a transverse scanning method, a double‐cladding single‐mode fiber (UVS‐INT‐PREMIUM, 100536, CorActive High‐Tech Inc.) was tapered down micrometer scale diameter (Figure S1a,b, Supporting Information) as follows: With a 5 mm width flame scanning across, the fiber was slowly stretched with two linear stages. The fiber‐optic geometric parameters such as diameter and length of the transition region were mainly determined by the moving speeds of the flame and stages (Figure S2, Supporting Information). A 10.5 µm diameter and 1.3 cm length uniform region was fabricated on the taper with a transition region of 0.3 cm in length (Figure S1b, Supporting Information).

The intermodal coupling of transition regions could be achieved for light propagating over the abrupt tapered region. Due to the relatively large index contrast between the core and cladding, the microfiber functioned as a Mach–Zehnder interferometer,^51^ allowing well separation of the fundamental mode (HE_11_) and higher‐order mode (HE_12_) to produce the π phase shift easily in the microfiber (Figure S3, Supporting Information). Normally, the external RI sensitivity of interferometer can be expressed by Equation (2), 23

(2) $$\frac{d\lambda }{dn_{\mathrm{sur}}}\;=\;\lambda \;\cdot \;\frac{1}{\Gamma }\;\cdot \;\left(\frac{1}{\Delta n_{\mathrm{eff}}}\;\frac{\partial \Delta n_{\mathrm{eff}}}{\partial n_{\mathrm{sur}}}\right)$$

where $\Gamma \;=\;1\;-\;\frac{\lambda }{\Delta n_{\mathrm{eff}}}\;\cdot \;\frac{d\Delta n_{\mathrm{eff}}}{d\lambda }$, the *n*_sur_ indicates the refractive index of fiber surface, Δ*n*_eff_ represents the effective index differences between the HE_11_ mode and HE_12_ mode, Γ is the dispersion factor, which can characterize the fluctuation of modal effective indices with the wavelength shift. In silica tapered microfiber with the diameter of around 10 µm, the Γ is negative. In Equation (2), with the increase of refractive index of fiber surface, the ∂Δ*n*_eff_/∂*n*_sur_ is normally negative because the high‐order mode of HE_12_ can have a greater increment than that of the HE_11_ mode.^22^ Hence, the transmission dips of the spectrum redshift with increasing RI of fiber surface.

*Materials and Reagents*: All chemicals and solvents supplied by Sigma‐Aldrich were analytical grade and were used without further purification. Tris(hydroxymethyl)aminomethane (Tris buffer, pH 7.4, 1.0 m), cyt c, human IgG, BSA, and the ssDNA (5′‐SH‐CCC TGT CTG GGG CCG ACC GGC GCA TTG GGT ACG TTG TTG C‐3′) aptamer were purchased from Sangon Inc. (Shanghai, China). The RGO and Ag@RGO nanosheets were obtained from XF Nano Inc. (Nanjing, China). The living B16‐F10 cells were cultivated and afforded by Dr. X. Ji at the Life Science and Technology Institute of Jinan University.

The cyt c solution was diluted with Tris buffer to concentrations from 10^−5^ m down to 10^−18^ m, respectively, for detection. The ssDNA aptamer was dissolved to form concentration of 10^−4^ m.

In the selectivity validation, the BSA, IgG, urea, glucose, and K^+^ solutions were diluted to a concentration of 10^−6^ m, respectively.

The mouse melanoma cells (B16‐F10) were supplied by Dr. X. Ji of College of Life Science and Technology at Jinan University.

*Nanointerface Functionalization of Silica Microfiber*: The silica microfiber was hydroxylated in a bath with piranha solution consisting of 30% H_2_O_2_ and concentrated H_2_SO_4_ at a volume ratio of 1:3 for 30 min, and then immersed in a 5% solution of APTES (99%) in ethanol for 40 min followed by a thorough wash with ethanol three times and with water for 30 min sequentially.^14^ The aminated microfiber was then immersed in the Ag@RGO (Figure S5a, Supporting Information) dispersion for 40 min before pulling out and drying in a vacuum. The according transmission spectrum wavelength shift was recorded in Figure 2e. A redshift of ≈2.5 nm was observed after the functionalization of 15 min and tended to be steady. It proved the successful functionalization of Ag@RGO nanointerface on silica microfiber surface, supplemented by SEM image in Figure S2c (Supporting Information).

In the controlling experiment, microfiber with RGO nanointerface was functionalized by the above approach by employing RGO (Figure S5b, Supporting Information) instead of Ag@RGO nanosheets.

*Immobilization of Aptamer on Microfiber*: The microfiber functionalized by Ag@RGO nanointerface was immersed in the aptamer solution for 70 min, and the π–π interaction between aptamer and Ag@RGO nanointerface was utilized to immobilize the aptamer on microfiber surface. The according transmission spectrum wavelength shift was recorded in Figure S8a (Supporting Information). A redshift of ≈3 nm was observed after the functionalization of 53 min and tended to be steady. It proved the successful immobilization of aptamer on microfiber surface, supplemented by SEM image in Figure S8b (Supporting Information).

*In the Control Experiments*: The aptamer immobilization on microfiber with RGO nanointerface was carried out by the above approach by employing microfiber with RGO nanointerface instead of one with Ag@RGO. The aptamer immobilization on microfiber without interface was carried out on the aminated microfiber surface through the electrostatic attraction between amino groups on microfiber and thiol groups of aptamer.

*Characterization*: The morphology of the Ag@RGO and RGO nanosheets was observed by a transmission electron microscopy (JEOL, JEM‐2100F). The surface morphology of microfiber was observed by an SEM (Hitachi, S4800) and an AFM (Bioscope Catalyst Nanoscope‐V). The analysis of the elementary distribution was performed on an EDX detector (EMAX) and X‐ray photoelectron spectroscopy. The surface composition information of the sensor was recorded using a micro Raman spectrometer (Thermo Fisher Scientific, DXR, excited by 532 nm laser line). The optical microscope photos of cells were taken by an optical microscope (ZEISS, Observer. A1).

*Experimental Setup*: The microfiber sensor was fixed in a microchannel (width 1 mm × height 500 µm) with the help of UV‐sensitive adhesive both sides over the sensing element of centimeter in length. This experimental setup protected the stability of sensor and biotesting.

The microfiber was excited by a broadband source (BBS) with light over 1500–1600 nm range, and the average light intensity of the BBS was over the range from −20 to −15 dBm nm^−1^ (working at a low‐level average power). The spectrum was monitored by an optical spectrum analyzer with a minimum wavelength resolution of 0.02 nm.

## Conflict of Interest

The authors declare no conflict of interest.

## Supporting information

Supplementary
