# Supplementary material for: Real‐Time Cellular Cytochrome C Monitoring through an Optical Microfiber: Enabled by a Silver‐Decorated Graphene Nanointerface
Source: Adv Sci (Weinh). 2018 Jun 7;5(8):1701074. doi: 10.1002/advs.201701074 (PMC6096990; doi:10.1002/advs.201701074)
Supplement: Supplementary file 1 — Supplementary [file ADVS-5-1701074-s001.pdf]

Copyright WILEY-VCH Verlag GmbH & Co. KGaA, 69469 Weinheim, Germany, 2018.

ADVANCED SCIENCE
Open Access

Supporting Information

for *Adv. Sci.,* DOI: 10.1002/advs.201701074

Real-Time Cellular Cytochrome C Monitoring through an Optical Microfiber: Enabled by a Silver-Decorated Graphene Nanointerface

Hongtao Li, Yunyun Huang, Chaoyan Chen, Aoxiang Xiao, Guanhua Hou, Yugang Huang, Xinhuan Feng, and Bai-Ou Guan**

Copyright WILEY-VCH Verlag GmbH & Co. KGaA, 69469 Weinheim, Germany, 2016.

Supporting Information

Real-Time Cellular Cytochrome C Monitoring through an Optical Microfiber: Enabled by a Silver-Decorated Graphene Nanointerface

Hongtao Li, Yunyun Huang, Chaoyan Chen, Aoxiang Xiao, Guanhua Hou, Yugang Huang, Xinhuan Feng, Bai-Ou Guan**

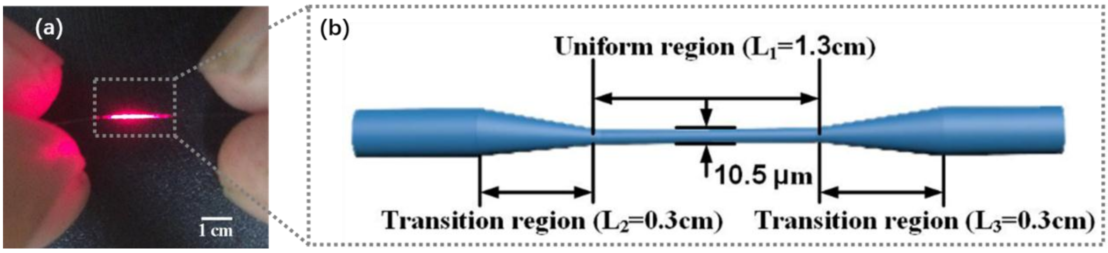

Figure S1. a) Photo of the silica microfiber. b) Schematic geometry of fabricated tapered silica microfiber interferometer.

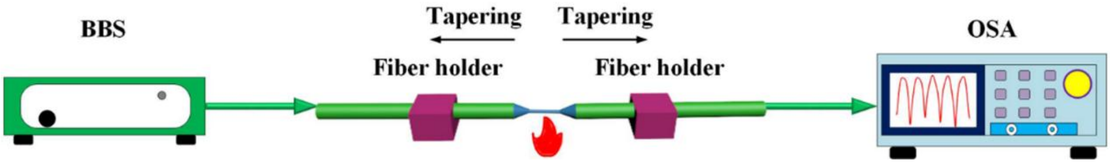

Figure S2. Creating platform of silica microfiber interferometer.

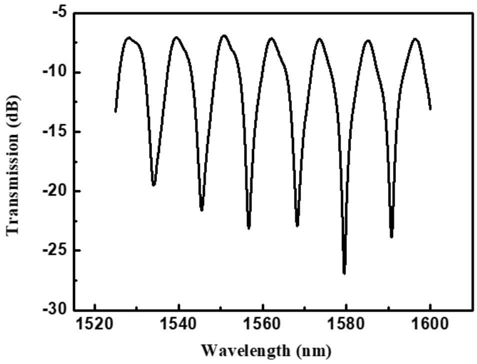

Figure S3. The transmission spectrum of silica optical microfiber

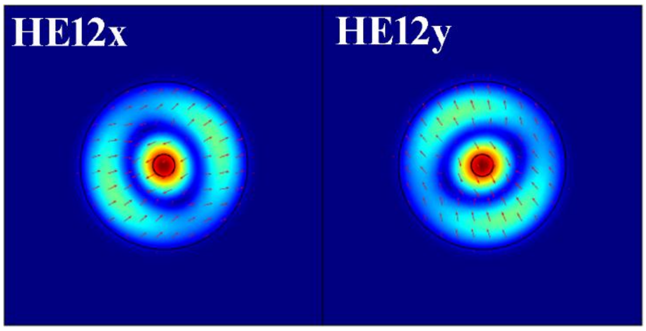

Figure S4. The composition of the reflective resonances and the transverse electric field amplitude distribution of the HE_{12} mode of the silica tapered microfiber.

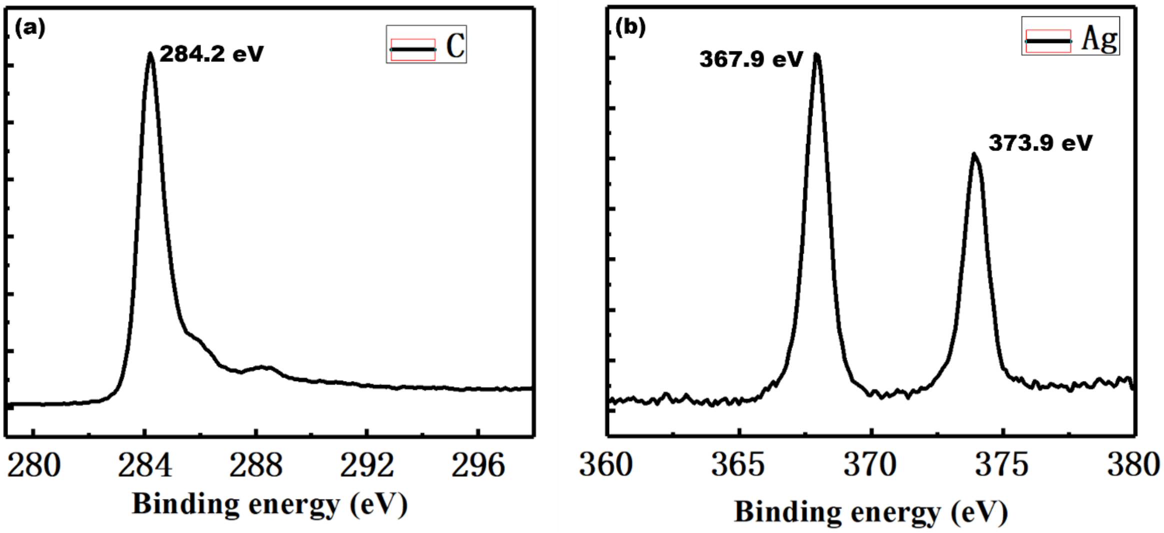

Figure S5. XPS profiles of Ag@RGO nanosheets.

The X-ray photoelectron spectroscopy (XPS) C1s core level spectrum of Ag@RGO clearly indicates that the structure of Ag@RGO with low oxygen content and a large number of sp^2 C=C bonds, exhibiting a high degree of reduced GO has been achieved.[26] In Figure R5b, the Ag $3d_{5/2}$ peak is located at 367.9 eV, and the Ag $3d_{3/2}$ is found at 373.9 eV. The slitting of the 3d doublet of Ag is 6.0 eV, indicating the formation of metallic silver on RGO nanosheets.[27, 28] This result proves that the silver nanoparticles were bonded with the RGO nanosheets.

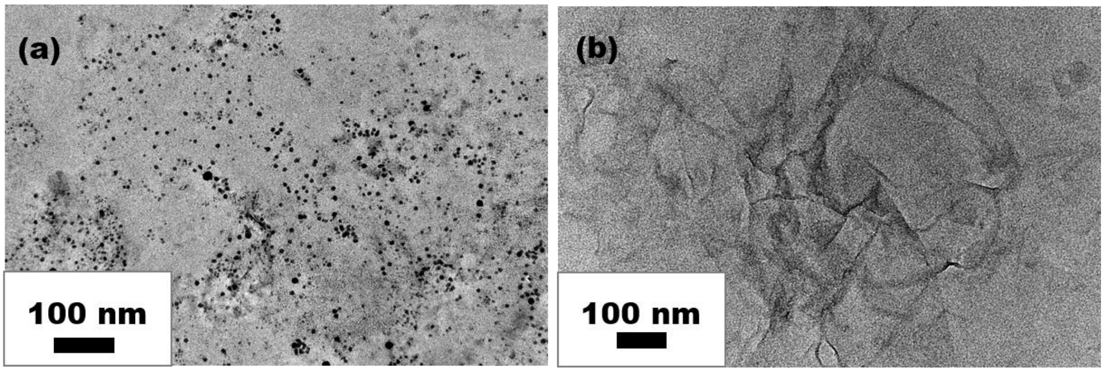

Figure S6. TEM image of a) Ag@RGO nanosheets and b) GO nanosheets.

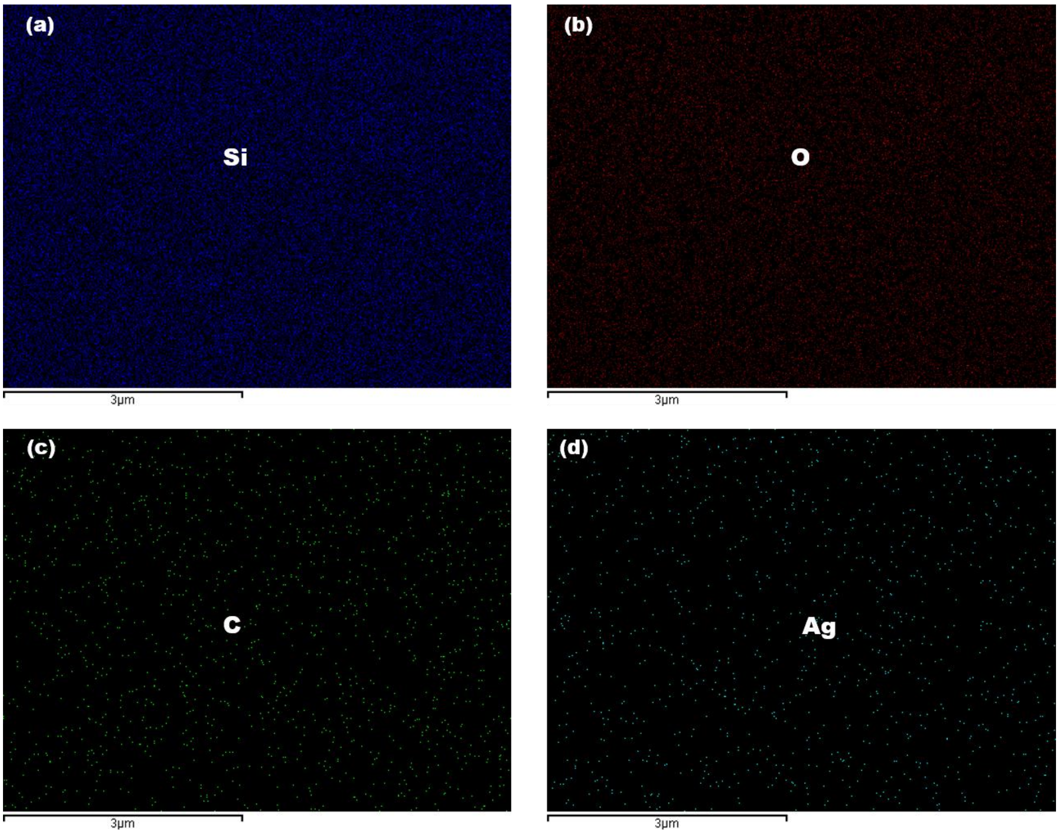

Figure S7. EDX mapping images of (a) Si, (b) O, (c) C and (d) Ag elements on the microfiber with Ag@RGO nanointerface.

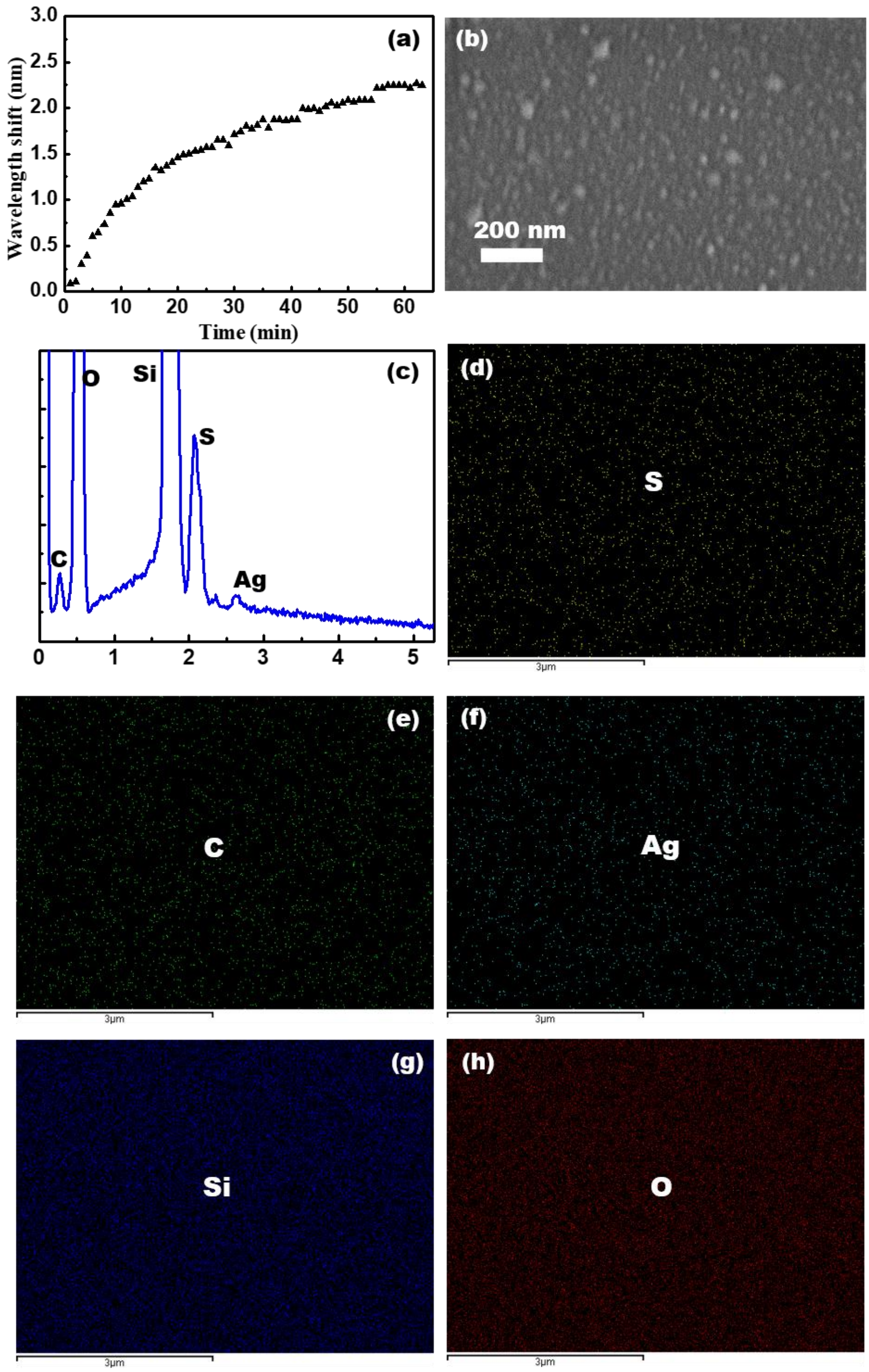

Figure S8. (a) The wavelength shift of interferometer in the aptamer immobilization process on Ag@RGO functionalized microfiber and (b) the SEM image and (c) EDX of microfiber with Ag@RGO nanointerface and aptamer, and the corresponding EDX mapping images of (d) S, (e) C, (f) Ag, (g) Si and (h) O elements.

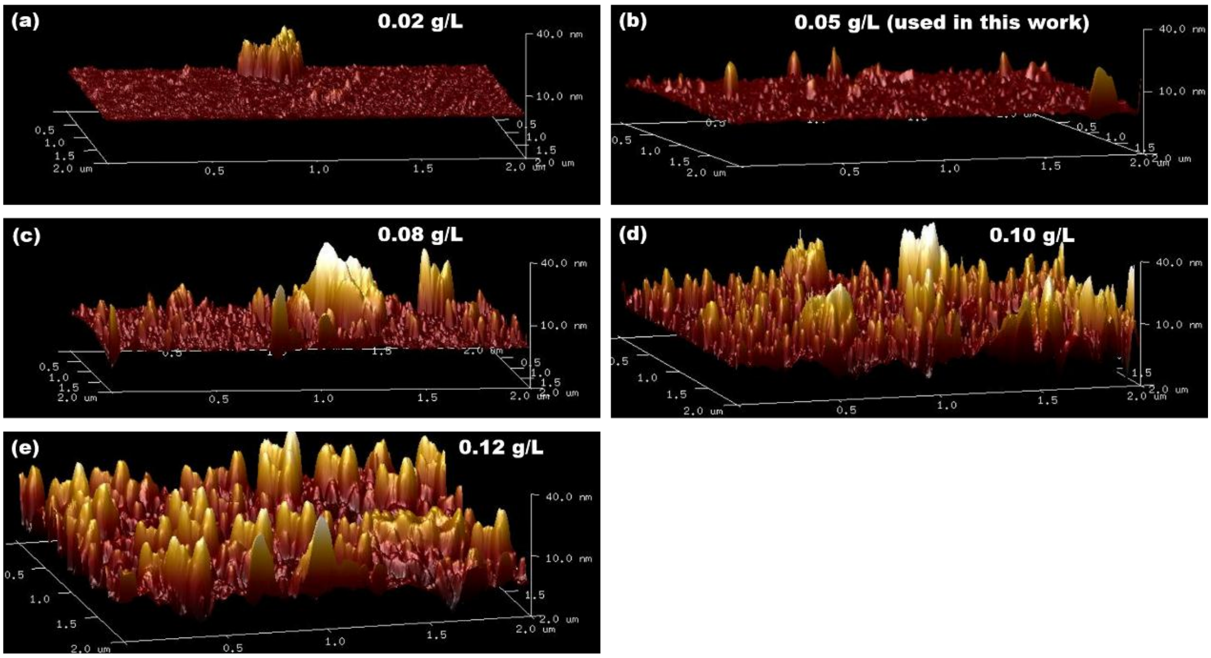

Figure S9. AFM image of the microfiber surface functionalized by Ag@RGO (by immersed in the Ag@RGO dispersion with different concentrations: a) 0.02 g/L; b) 0.05 g/L; c) 0.08 g/L; d) 0.10 g/L; e) 0.12 g/L)

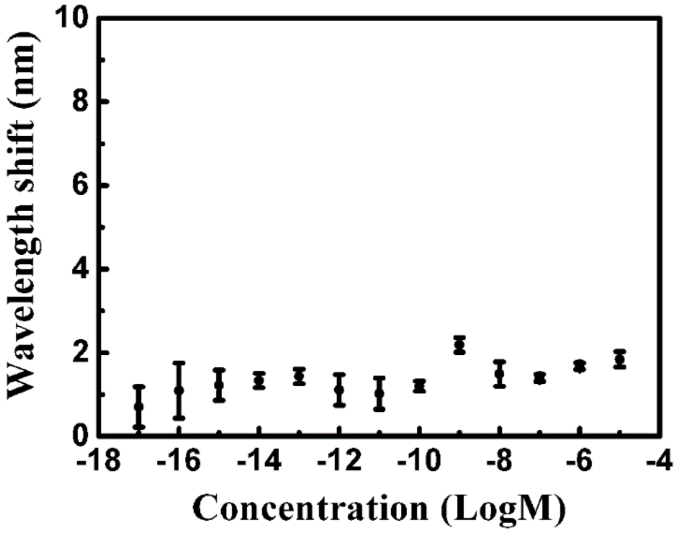

Figure S10. The wavelength shift of the bare tapered silica microfiber interferometer versus the concentrations of cyt c.
